# A Novel Frequency Stabilization Approach for Mass Detection in Nonlinear Mechanically Coupled Resonant Sensors

**DOI:** 10.3390/mi12020178

**Published:** 2021-02-11

**Authors:** Lei Li, Hanbiao Liu, Mingyu Shao, Chicheng Ma

**Affiliations:** 1 School of Transportation and Vehicle Engineering, Shandong University of Technology, Zibo 255049, China; liuhanbiaotongxue@163.com (H.L.); shaomingyu@sdut.edu.cn (M.S.); machch@sdut.edu.cn (C.M.); 2 State Key Laboratory of Mechanical System and Vibration, School of Mechanical Engineering, Shanghai Jiao Tong University, Shanghai 200240, China

**Keywords:** MEMS, nonlinear dynamics, coupled vibrations, bifurcation

## Abstract

Frequency stabilization can overcome the dependence of resonance frequency on amplitude in nonlinear microelectromechanical systems, which is potentially useful in nonlinear mass sensor. In this paper, the physical conditions for frequency stabilization are presented theoretically, and the influence of system parameters on frequency stabilization is analyzed. Firstly, a nonlinear mechanically coupled resonant structure is designed with a nonlinear force composed of a pair of bias voltages and an alternating current (AC) harmonic load. We study coupled-mode vibration and derive the expression of resonance frequency in the nonlinear regime by utilizing perturbation and bifurcation analysis. It is found that improving the quality factor of the system is crucial to realize the frequency stabilization. Typically, stochastic dynamic equation is introduced to prove that the coupled resonant structure can overcome the influence of voltage fluctuation on resonance frequency and improve the robustness of the sensor. In addition, a novel parameter identification method is proposed by using frequency stabilization and bifurcation jumping, which effectively avoids resonance frequency shifts caused by driving voltage. Finally, numerical studies are introduced to verify the mass detection method. The results in this paper can be used to guide the design of a nonlinear sensor.

## 1. Introduction

Recently, mass detection of very small chemical and biological species using microelectromechanical systems (MEMS) has attracted wide attention [1,2,3,4,5,6]. A MEMS mass sensor mainly uses the mechanical characteristics changes before and after the adsorption mass of a resonant element to detect and identify the target analyte [7]. Mass-sensing methods based on a frequency shift [8] have gained increasing attention, as these methods maintain the quasi-digital [9] nature of the signal. One challenge associated with these methods is that in the nonlinear vibration region, the resonance frequency depends heavily on vibration amplitude [10,11,12], which affects the accuracy of the mass sensor. In this article, a mechanically coupled resonant structure is designed to realize the frequency stabilization in the nonlinear regime. In addition, a quantitative relationship between resonance frequency and added mass is proposed by using frequency stabilization, which can avoid the dependence of resonant frequency on amplitude and greatly improve the mass detection accuracy.

MEMS mass sensors are often affected by the typical nonlinear electrostatic forces, geometric nonlinearity, environmental interference, and other factors [13,14,15]. This can bring about an undesirable interdependence between the resonance frequency and the vibration amplitude, which is obviously disadvantageous for MEMS mass sensors. It is discovered that the frequency–amplitude interdependence can be drastically reduced when appropriate coupled-mode vibration exists in a nonlinear system [16]. Mode coupled vibration is a universal phenomenon, where multiple vibration modes transfer energy to each other due to the coupling [17]. Coupled-mode vibration can be divided into two types: One is the vibration energy transfer between different structures caused by mechanical components; the other is the vibration energy transfer between different modes within the structure caused by electric field forces and geometric nonlinearity [18,19]. Kilinc et al. [20] designed a nanoarray coupling resonant structure and obtained complex coupling resonance behavior by adjusting the coupling stiffness between different resonators. Li et al. [21] presented coupled vibration behavior between second-order and third-order modes caused by the axial stress, which can be used to realize high precision parameter identification by an antisymmetric mode. In addition, weakly coupled nonlinear MEMS resonators can lead to mode localization, which can greatly improve the sensitivity of the sensor [22]. Antonio et al. [23] used coupled-mode vibration to propose a novel method in order to realize frequency stabilization by coupling two different vibration modes. It was discovered that the low-frequency noise resonator was possible in a nonlinear regime. Zhang et al. [24] experimentally studied the vibration energy transfer between the flexural mode and the extensional bulk mode of a cantilever beam resonator by exciting the two modes simultaneously. Experimental results showed that the interdependence between the resonance frequency and the vibration amplitude can be limited by the mode interaction in nonlinear micromechanical resonators [25]. Zanette [26] studied the joint dynamics of coupled Duffing oscillators with a nonlinearity of opposite signs. Results showed that the frequency stabilization of nonlinear coupled systems can be achieved under appropriate parameter conditions. As it is known, nonlinear factors can lead to complex dynamic phenomena such as bifurcation and chaos, which can seriously affect the dynamic performance of the mass sensor [27]. Tchakui et al. [28] studied the dynamic bifurcation behaviors of the unidirectionally coupled nonlinear electromechanical systems considering three situations. Various dynamical behaviors corresponding to different types of bifurcation were obtained with varying the coupling coefficient. In recent years, many researchers have analyzed coupling resonance behaviors to reveal the complex dynamic bifurcations and to improve frequency stabilization of nonlinear systems [29,30,31,32,33,34,35].

A MEMS resonant mass sensor mainly realizes detection by changing the resonant frequency and vibration amplitude of the structure caused by the adsorption of the elastic element of the sensor to the target analyzers [36,37,38,39]. Bouchaala et al. [40] derived analytical formulations to calculate the induced resonance frequency shifts caused by an added mass. The results indicated that the detection sensitivity increases with the decrease of size. However, with the reduction of size and complexity of the structure, there are obvious nonlinear effects and complex bifurcation behaviors [41]. Younis et al. [42] utilized the dynamic instabilities and bifurcations in a MEMS to realize novel methods and functionalities for mass detection. It was noted that bifurcation-based mass detection methods led to dramatically enhanced sensitivity and less performance deterioration due to measurement noise as compared to frequency shift-based methods [43]. Similarly, Nguyen et al. [44] used bifurcation jumping characteristics to propose a mass threshold detection method. Results showed that sudden jumps in amplitude can make the detection of a very small mass possible. Hasan et al. [45] studied the intelligent adjustable threshold pressure switch. When the pressure exceeds the critical threshold, the system can be induced to produce an amplitude jump, realizing the rapid sensing of pressure value. However, most of the bifurcation-based mass sensors are used in mass threshold detection. It is difficult to achieve high precision quantitative mass detection using this method.

It can be concluded from the above research status that frequency stabilization and mass detection performance are very important in the design of MEMS mass sensors and should be taken into account [46,47]. However, to the best of our knowledge, the physical conditions and influencing factors of frequency stabilization have not been systematically investigated. In addition, an effective parameter identification method is needed to realize an accurate detection of mass sensors operating in a nonlinear vibration range. The dependence of resonance frequency on amplitude seriously affects the mass detection results. In this paper, we design a mechanically coupled structure to realize frequency stabilization by improving the quality factor and adjusting the driving voltage, which overcomes the effect of voltage fluctuation on resonance frequency and improves the robustness of nonlinear sensor. Then, a novel approach for mass detection in a nonlinear coupled resonant sensor based on frequency stabilization and bifurcation jumping is proposed, which greatly improves the performance of the nonlinear sensor.

The structure of this article is as follows. In Section 2, Hamilton’s principle and Galerkin discretization are introduced to obtain governing equations. In Section 3, two different vibration modes are coupled through the internal resonance, which can stabilize the oscillation frequency of the nonlinear system. In Section 4, the influence of voltage fluctuation on dynamic behavior is studied. In Section 5, a novel approach for mass detection in a nonlinear coupled resonant sensor is proposed. Finally, summary and conclusions are presented in the last section.

## 2. Problem Formulation

**Figure 1:**
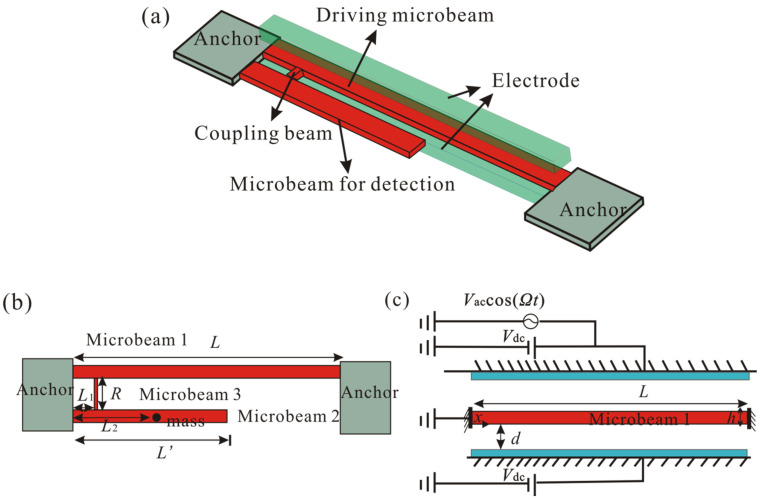
Schematic diagram of the mechanically coupled resonance sensor. (**a**) Three-dimensional schematic diagram of the resonant structure; (**b**) size parameters of the resonant mass sensor; (**c**) schematic diagram of the electrically actuated microbeam structure.

An electrically actuated microbeam structure has important applications in MEMS mass sensors [1]. The mass added to the microbeam can result in downshifting its natural frequency. However, the electrostatic and geometric nonlinearity can lead to a dependence of resonant frequency on amplitude. To provide a stable resonance frequency, a mechanically coupled resonant element was designed, as shown in Figure 1. A thin coupling beam (microbeam 3) was introduced to realize vibration coupling between microbeams 1 and 2. The coupling strength can be controlled through the position, moment of inertia, and length of the coupling beam. Microbeam 1 is a doubly clamped microresonator driven by means of a pair of bias voltages and an AC voltage component, as shown in Figure 1c. Microbeam 2 is a cantilever resonator driven by the coupling beam. Size parameters and physical properties of the system are listed in Table 1. Then, a lumped mass *m* is added at $x=L_{2}$, as shown in Figure 1b. Added mass can be detected by observing the resonance frequency of the device. Because the mass of the coupling beam is much less than that of the resonators, the coupling beam can be assumed to be a spring that mechanically couples resonators with each other. When the system oscillates slightly around the equilibrium point, the interaction between the two oscillators is assumed to be linear and the stiffness of the coupling beam can be obtained by the finite element software. Then, by using Hamilton’s principle, the equations of motion that govern the transverse deflections ${\hat{w}}_{1}(\hat{x},\hat{t})$ and ${\hat{w}}_{2}(\hat{x},\hat{t})$ are written as

(1) $$\begin{array}{l} \rho A\frac{\partial^{2}{\hat{w}}_{1}}{\partial {\hat{t}}^{2}}+EI{\hat{w}}_{1}^{iv}+c_{1}\frac{\partial {\hat{w}}_{1}}{\partial \hat{t}}=(\frac{EA}{2L}\int_{0}^{L}{{\hat{w}}_{1}^{\prime }}^{2}dx){\hat{w}}_{1}^{\prime \prime }\\ +\frac{\varepsilon_{0}b{\left[V_{dc}+V_{ac}\cos(\hat{\Omega }\hat{t})\right]}^{2}}{2{\left(d-{\hat{w}}_{1}\right)}^{2}}-\frac{\varepsilon_{0}bV_{dc}^{2}}{2{\left(d+{\hat{w}}_{1}\right)}^{2}}+k({\hat{w}}_{2}-{\hat{w}}_{1})\delta (x-L_{1}) \end{array}$$

(2) $$\left[\rho A+\delta (x-L_{2})m]\frac{\partial^{2}{\hat{w}}_{2}}{\partial {\hat{t}}^{2}}+EI{\hat{w}}_{2}^{iv}+c_{2}\frac{\partial {\hat{w}}_{2}}{\partial \hat{t}}=k({\hat{w}}_{1}-{\hat{w}}_{2})\delta (x-L_{1}\right)$$

The boundary conditions for the coupled resonance structure are

$${\hat{w}}_{1}^{\prime }(0,\hat{t})={\hat{w}}_{1}(0,\hat{t})={\hat{w}}_{1}(L,\hat{t})={\hat{w}}_{1}^{\prime }(L,\hat{t})=0$$

$${\hat{w}}_{2}^{\prime }(0,\hat{t})={\hat{w}}_{2}(0,\hat{t})={\hat{w}}_{2}^{\prime \prime }(L^{\prime },\hat{t})={\hat{w}}_{2}^{\prime \prime \prime }(L^{\prime },\hat{t})=0$$

where ${\hat{w}}_{i}^{\prime }=\frac{\partial {\hat{w}}_{i}}{\partial \hat{x}}$ for *i* =1, 2.

Here, $\hat{x}$ is the position along the beam length, *A* and *I* are the area and moment of inertia of the cross section, $L^{\prime }$ is the length of microbeam 2, and $\hat{t}$ is the time. The last terms in Equations (1) and (2) represent the elastic restoring force caused by the coupling beam, where *k* represents the coupling strength coefficient.

Galerkin discretization method is introduced to deal with partial differential equations. Then, considering the first and second vibration modes, we can obtain the governing equations:

(3) $$\begin{array}{l} \frac{d^{2}u_{1}}{dt^{2}}+c_{1n}\frac{du_{1}}{dt}+\beta_{1}^{2}u_{1}-[\alpha_{1}\int_{0}^{1}{\phi_{1}^{\prime }}^{2}dx\int_{0}^{1}\phi_{1}^{\prime \prime }\phi_{1}dx+8\alpha_{2}V_{dc}^{2}\int_{0}^{1}\phi_{1}^{4}dx]u_{1}^{3}\\ =2\alpha_{2}V_{dc}V_{ac}\int_{0}^{1}\phi_{1}dx\cos\Omega t+k^{\prime }[u_{2}\phi_{2}(l_{1})\phi_{1}(l_{1})-u_{1}\phi_{1}^{2}(l_{1})] \end{array}$$

(4) $$\left(1+\eta^{\prime })\frac{d^{2}u_{2}}{dt^{2}}+c_{2n}\frac{du_{2}}{dt}+\beta_{2}^{2}u_{2}=k^{\prime }[u_{1}\phi_{2}(l_{1})\phi_{1}(l_{1})-u_{2}\phi_{2}^{2}(l_{1})\right]$$

A detailed derivation and parameters are given in Appendix A.

**Figure 2:**
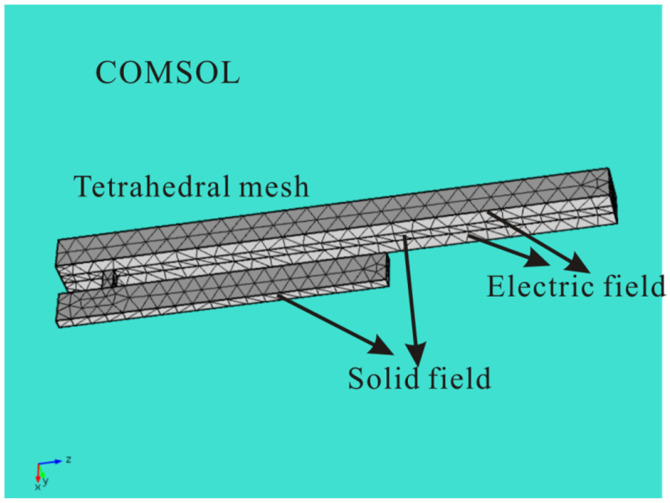
Finite element model of the coupled resonator obtained by the COMSOL software.

**Figure 3:**
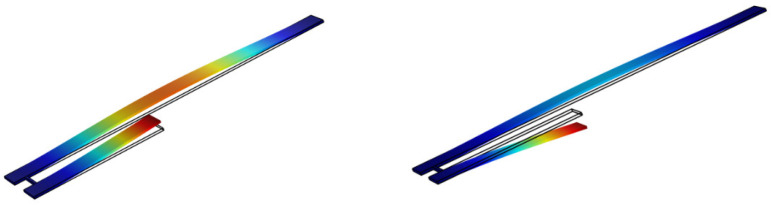
The first two vibration modes of the mechanically coupled structure in COMSOL.

To verify the theoretical model and obtain the vibration mode of the coupled resonator, the software COMSOL was introduced to study the coupled resonant structure by using the multifield solver [49], as shown in Figure 2. Here, the coupling strength coefficient *k* can be obtained by using the software COMSOL. To get the two lowest frequencies of the system to be as close as possible, the stiffness of the coupling beam should not be too large. Meanwhile, to ensure the energy transfer between different resonators, the coupling beam stiffness should not be too small. When the length of the cantilever is near 60 µm, we obtain the coupling strength coefficient k=507 N/m. Figure 3 shows the first mode and the second mode of the coupled structure. It was found that the first mode is in phase and the second mode is out of phase.

Through Equations (3) and (4), the Jacobian matrix for the linear system can be obtained:

(5) $$J=\left[\begin{array}{cc} \beta_{1}^{2}+k^{\prime }\phi_{1}^{2}(l_{1}) & -k^{\prime }\phi_{2}(l_{1})\phi_{1}(l_{1})\\ \frac{-k^{\prime }\phi_{2}(l_{1})\phi_{1}(l_{1})}{1+\eta^{\prime }} & \frac{\beta_{2}^{2}+k^{\prime }\phi_{2}^{2}(l_{1})}{1+\eta^{\prime }} \end{array}\right]$$

Then, the resonant frequencies can be obtained by solving the eigenvalues of Equation (5).

(6) $$(\beta_{1}^{2}+k^{\prime }\phi_{1}^{2}(l_{1})-\omega^{2})(\frac{\beta_{2}^{2}+k^{\prime }\phi_{2}^{2}(l_{1})}{1+\eta^{\prime }}-\omega^{2})-\frac{{\left(k^{\prime }\phi_{2}(l_{1})\phi_{1}(l_{1})\right)}^{2}}{1+\eta^{\prime }}=0$$

**Figure 4:**
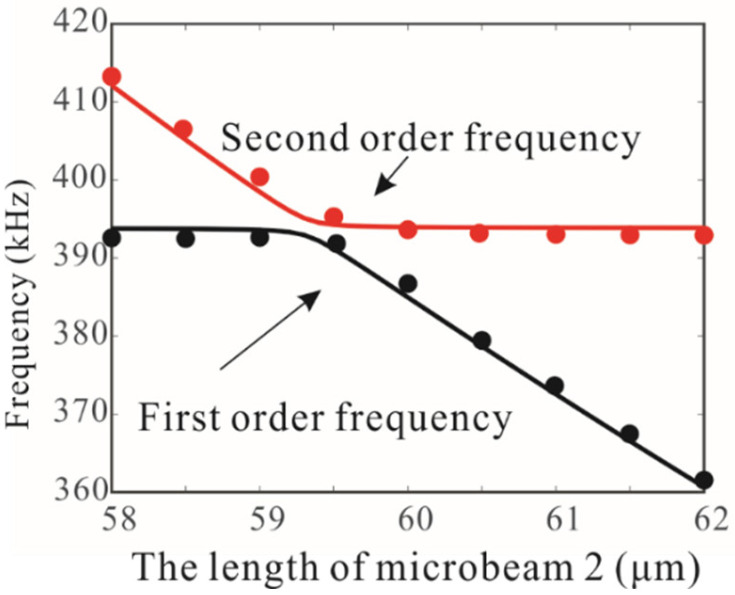
Variation of the first two natural frequencies versus the length of microbeam 2 when $V_{dc}=2\;V$. The lines denote the theoretical results and the points denote the COMSOL results [49].

Figure 4 shows the variation of the first natural frequency and the second natural frequency versus the length of microbeam 2 when $V_{dc}=2\;V$. The increase of the length of microbeam 2 can reduce the natural frequencies of the system. When the length of microbeam 2 is near 59.5 µm, the first natural frequency of the system is approximately equal to the second natural frequency. Then, 1:1 internal resonance may occur. Here, the results obtained by COMSOL are given to verify the theoretical results. The error between theoretical results and the COMSOL results is very small, which means that the theoretical method and equivalent coupling stiffness are reasonable. Following this, we focus on the frequency stabilization caused by internal resonance. It should be noted that the driving voltage selected in this paper is far less than the pull-in voltage.

## 3. Nonlinear Dynamic Behavior

In this section, the complex dynamic bifurcation behaviors in the weakly coupled system are considered.

### 3.1. Perturbation Analysis

To simplify Equations (3) and (4), they can be rewritten as

(7) $$\frac{d^{2}u_{1}}{dt^{2}}+c_{1n}\frac{du_{1}}{dt}+\omega_{1}^{2}u_{1}-\kappa u_{2}+k_{1a}u_{1}^{3}=f\cos\Omega t$$

(8) $$(1+\eta^{\prime })\frac{d^{2}u_{2}}{dt^{2}}+c_{2n}\frac{du_{2}}{dt}+\omega_{2}^{2}u_{2}=\kappa u_{1}$$

where $\kappa =k^{\prime }\phi_{2}(l_{1})\phi_{1}(l_{1})$, $f=2\alpha_{2}V_{dc}V_{ac}\int_{0}^{1}\phi_{1}dx$, $\omega_{1}^{2}=\beta_{1}^{2}+k^{\prime }\phi_{1}^{2}(l_{1})$, $\omega_{2}^{2}=\beta_{2}^{2}+k^{\prime }\phi_{2}^{2}(l_{1})$, and $k_{1a}=-\alpha_{1}\int_{0}^{1}{\phi_{1}^{\prime }}^{2}dx\int_{0}^{1}\phi_{1}^{\prime \prime }\phi_{1}dx-8\alpha_{2}V_{dc}^{2}\int_{0}^{1}\phi_{1}^{4}dx$.

Through Equation (8), we find that the vibration of microbeam 2 is linear. Firstly, we can use linear vibration theory to express the vibration form of microbeam 2 in terms of the displacement and velocity of microbeam 1.

We assume $c_{2n}=c_{1n}=c_{n}$ and obtain

(9) $$u_{2}=-\frac{\kappa c_{n}}{{\left(c_{n}\Omega \right)}^{2}+{\left(\omega_{2}^{2}-\Omega^{2}-\eta^{\prime }\Omega^{2}\right)}^{2}}\frac{du_{1}}{dt}-\frac{\kappa (\eta^{\prime }\Omega^{2}+\Omega^{2}-\omega_{2}^{2})}{{\left(c_{n}\Omega \right)}^{2}+{\left(\omega_{2}^{2}-\Omega^{2}-\eta^{\prime }\Omega^{2}\right)}^{2}}u_{1}$$

Substituting Equation (9) into Equation (7) yields the following:

(10) $$\frac{d^{2}u_{1}}{dt^{2}}+c^{\prime }\frac{du_{1}}{dt}+\omega_{n}^{2}u_{1}+k_{1a}u_{1}^{3}=f\cos\Omega t$$

Here, the equivalent damping and equivalent stiffness of microbeam 1 can be written as

(11) $$c^{\prime }=c_{n}-\kappa u_{s}$$

(12) $$\omega_{n}^{2}=\omega_{1}^{2}-\kappa u_{c}$$

where

(13) $$u_{s}=-\frac{\kappa c_{n}}{{\left(c_{n}\Omega \right)}^{2}+{\left(\omega_{2}^{2}-\Omega^{2}-\eta^{\prime }\Omega^{2}\right)}^{2}}$$

(14) $$u_{c}=-\frac{\kappa (\eta^{\prime }\Omega^{2}+\Omega^{2}-\omega_{2}^{2})}{{\left(c_{n}\Omega \right)}^{2}+{\left(\omega_{2}^{2}-\Omega^{2}-\eta^{\prime }\Omega^{2}\right)}^{2}}$$

A perturbation analysis is introduced to deal with Equation (10). Then, the amplitude–frequency response equation of the coupled resonator can be obtained.

(15) $$(\frac{1}{2}c^{\prime }{)}^{2}+{\left(\sigma -\frac{\lambda }{\omega_{n}}a^{2}\right)}^{2}={\left(\frac{f}{2\omega_{n}a}\right)}^{2}$$

The detailed derivation and parameters are given in Appendix B.

### 3.2. Coupled-Mode Vibration

**Figure 5:**
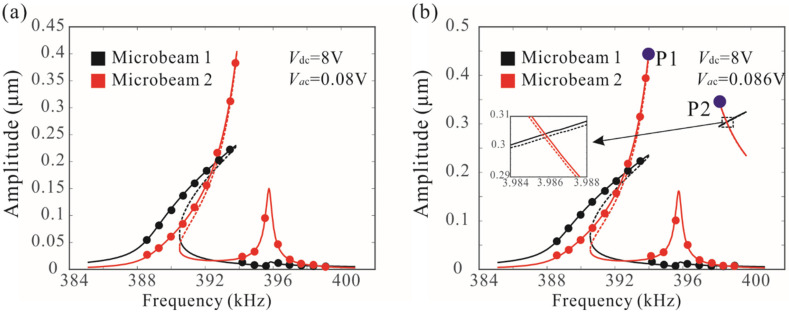
(**a**,**b**) Combined frequency response curves of the mechanically coupled microbeam resonators when $L^{\prime }=59.2\;\mu m$. The lines denote the theoretical results, and the points denote the numerical results; P1 and P2 represent peak frequencies.

**Figure 6:**
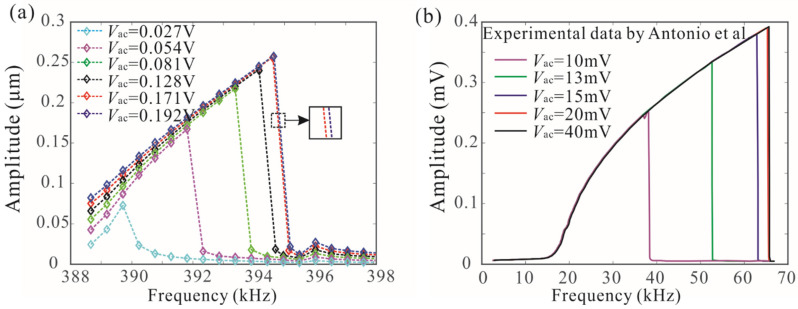
Frequency stability phenomena of mechanically coupled resonators. (**a**) Amplitude resonance curves of microbeam 1 with $V_{dc}=8\;V$ and different AC values; (**b**) experimental results of coupled resonant structure obtained by Antonio et al. [23].

Figure 5 shows the nonlinear coupled mode vibration behavior of a resonator under different AC voltages. When $V_{ac}=0.08\;V$, there is typically hard nonlinearity in the amplitude–frequency response curves. The resonant frequency of the system depends heavily on the amplitude. As the AC voltage increases, it was discovered that the discontinuous phenomenon of frequency response curve occurs, which can lead to the isolated branches, as shown in Figure 5b. The long-time integration of Equations (3) and (4) is introduced to verify the analytical solution derived from the method of multiple scales. To further analyze the complex dynamic behavior, the swept harmonic responses of microbeam 1 are obtained by sweeping up the frequency under different AC voltages, as shown in Figure 6a. For $V_{ac}<0.171\;V$, the peak frequency increases with driving force, but for the higher driving voltages, the peak frequency changes slightly. It was found that this discontinuity phenomenon caused by coupled-mode vibration can improve the stability of the peak frequency, which has important potential applications in mass sensors. In addition, the frequency stabilization caused by coupling modes has been experimentally studied, as shown in Figure 6b [23]. The experimental results are qualitatively consistent with the theoretical results in this paper.

### 3.3. Frequency Stabilization

Frequency stabilization is caused by the energy transfer between different vibration modes in a weakly coupled system. We propose theoretically the physical conditions for frequency stabilization and analyze the influence of quality factor on frequency stabilization. Firstly, we try to obtain an analytical expression for the resonance frequency. The backbone curve can be obtained by Equation (15):

(16) $$a_{1}^{2}(\Omega )=\frac{\sigma \omega_{n}}{\lambda }$$

When the vibration amplitude of microbeam 1 is equal to that of the backbone curve, the resonance of the system occurs. Substituting Equation (16) into Equation (15) yields the following:

(17) $$\frac{\lambda f^{2}}{\sigma \omega_{n}}-{\left(c^{\prime }\omega_{n}\right)}^{2}=0$$

Substituting Equations (11) and (12) into Equation (17) yields the following:

(18) $$\frac{\lambda f^{2}}{\sigma \sqrt{\left(\omega_{1}^{2}-\kappa u_{c}\right)}}-{\left(c_{n}-\kappa u_{s}\right)}^{2}(\omega_{1}^{2}-\kappa u_{c})=0$$

As a function of *f*, Ω is the root of a nonlinear polynomial equation, with one or three real positive solutions. Working out their analytical expressions is impractical, but accurate approximations can be obtained when the quality factor is high enough. Through Figure 5 and Figure 6, it was found that the frequency stabilization occurs when $\Omega \approx \omega_{2}/\sqrt{1+\eta^{\prime }}$ (i.e., the natural frequency of microbeam 2). Thus, $u_{c}\approx 0$ is obtained by Equation (14). Then, substituting $u_{c}=0$, $\Omega =\omega_{n}+\varepsilon \sigma$ and Equation (13) into Equation (18) yields the following:

(19) $$\frac{\lambda f^{2}}{(\Omega -\omega_{1})\omega_{1}}-{\left[c_{n}+\frac{\kappa^{2}c_{n}}{{\left(c_{n}\Omega \right)}^{2}+{\left(\omega_{2}^{2}-\Omega^{2}-\eta^{\prime }\Omega^{2}\right)}^{2}}\right]}^{2}\omega_{1}^{2}=0$$

To indicate the resonant frequency near $\omega_{2}/\sqrt{1+\eta^{\prime }}$, Equation (19) can be rewritten as

(20) $$\Omega =\frac{\omega_{2}}{\sqrt{1+\eta^{\prime }}}\pm \frac{c_{n}}{2(1+\eta^{\prime })}\sqrt{\frac{(1+\eta^{\prime })\kappa^{2}\omega_{1}/c_{n}\omega_{2}^{2}}{\sqrt{\lambda f^{2}/(\omega_{2}/\sqrt{1+\eta^{\prime }}-\omega_{1})\omega_{1}}-c_{n}\omega_{1}}-1}$$

The detailed derivation is given in Appendix C.

**Figure 7:**
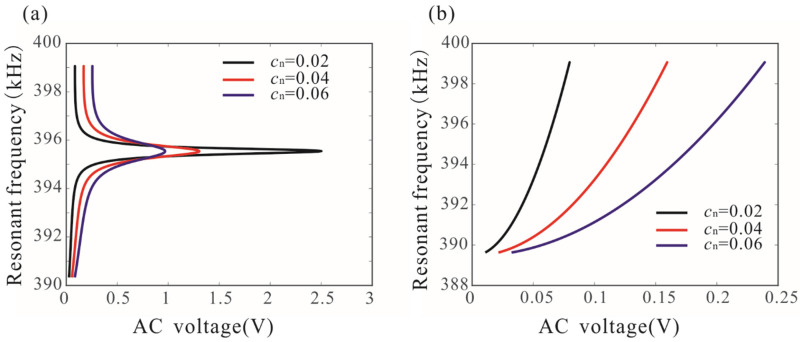
Variation of the resonance peak frequencies of the system versus the alternating current (AC) voltages under different damping. Here, (**a**) represents coupled resonant structure and (**b**) represents the single-degree-of-freedom resonant structure when κ=0.

Two solutions to Equation (20) represent the two peak frequencies (P1 and P2 as shown in Figure 5b) of the resonant system. Figure 7a shows the effects of damping and driving voltage on the peak frequencies. It was found when the driving force is below the critical value, the peak frequency of the system increases significantly with the increase of the driving voltage. When the driving voltage is in a certain range, the peak frequency has very little dependence on the driving voltage, which is called frequency stabilization. Improving the quality factor is beneficial to improving the frequency stabilization of the system. Here, the resonance peak frequency of a single-degree-of-freedom system is also obtained under different AC voltages, as shown in Figure 7b. Due to nonlinearity, the resonance peak frequency of the system depends heavily on the driving voltage. There is no frequency stabilization for a nonlinear system with a single degree of freedom.

Then, the physical conditions for frequency stabilization can be obtained from Figure 7. By Equation (20), it was found that the peak frequency reaches the upper critical value when $(1+\eta^{\prime })\kappa^{2}\omega_{1}/c_{n}\omega_{2}^{2}=\sqrt{\lambda f^{2}/(\omega_{2}/\sqrt{1+\eta^{\prime }}-\omega_{1})\omega_{1}}-c_{n}\omega_{1}$. Then, we can obtain

(21) $$f_{\max}=\frac{(1+\eta^{\prime })\kappa^{2}\omega_{1}/c_{n}\omega_{2}^{2}+c_{n}\omega_{2}}{\sqrt{\lambda /(\omega_{2}/\sqrt{1+\eta^{\prime }}-\omega_{1})\omega_{1}}}$$

Through Figure 7, when the resonance frequency of a single-degree-of-freedom system is equal to $\omega_{2}/\sqrt{1+\eta^{\prime }}$, frequency stabilization appears in the coupling system. Considering κ=0, we can obtain the minimum critical voltage by Equation (17):

(22) $$f_{\min}=c_{n}\omega_{1}\sqrt{\frac{\omega_{1}(\omega_{2}/\sqrt{1+\eta^{\prime }}-\omega_{1})}{\lambda }}$$

**Figure 8:**
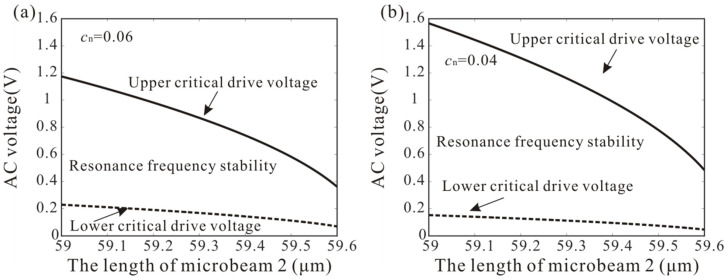
(**a**,**b**) Variation of the parameter space of frequency stabilization versus the length of microbeam 2 and AC voltage. Solid lines represent upper critical drive voltage; dotted lines represent lower critical drive voltage.

When the driving force satisfies $f\in [f_{\min},f_{\max}]$, frequency stabilization occurs. Figure 8 shows the variation of frequency stabilization versus the length of microbeam 2 and AC voltage. As the length of microbeam 2 increases, the resonance frequency of the cantilever beam decreases. There is a positive correlation between the resonance frequency and the driving voltage. Thus, the lower critical drive voltage and the upper critical drive voltage of the frequency stabilization decreases. In addition, it was found that reducing damping can improve the frequency stabilization of the nonlinear system.

It is worth mentioning that the drive voltage has a slight effect on the peak frequency when frequency stabilization occurs. The performance of frequency stabilization is determined by the quality factor. When the quality factor of the system is high enough, the effect of the driving voltage on the peak frequency can be ignored. On the contrary, when the quality factor is low, there is no frequency stabilization in the coupled resonant system.

## 4. Robust Analysis

Environmental disturbances and voltage noise are important factors that affect the dynamic stability of the resonant sensor. Because the nonlinear resonant sensor has many stable periodic solutions, the basin of attraction of periodic solutions is very important to the dynamic performance of the sensor. Nguyen et al. [44] studied the robustness of mass detection mechanism and obtained the basin of attraction of periodic solutions under different design parameters. In addition, voltage noise is also very important to the stability of the sensor. In this section, we propose the influence mechanism of voltage noise on bifurcation frequency. The significance of frequency stabilization is to suppress the influence of voltage on system response. Voltage fluctuation was introduced to study the dynamic behavior and peak frequency of the coupled system. Equation (10) can be rewritten as

(23) $$\frac{d^{2}u_{1}}{dt^{2}}+c^{\prime }\frac{du_{1}}{dt}+\omega_{n}^{2}u_{1}+k_{1a}u_{1}^{3}=(f+\xi (t))\cos\Omega t$$

where the fluctuating part ξ(t) represents the noise produced by random modulations of the driving force, which is caused by the amplitude fluctuation of AC voltage. We consider the voltage fluctuation ξ(t) to be white Gaussian noise. Then, a random sequence with a mean of 0 and a variance of 1 is generated in MATLAB to study the influence of voltage fluctuation on dynamic behavior.

**Figure 9:**
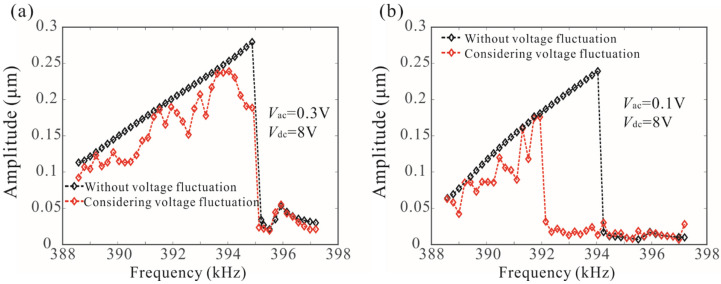
Effect of voltage fluctuation on dynamic behavior of a coupled resonance system. (**a**) The voltage fluctuation has no effect on the resonance frequency when $V_{ac}=0.3\;V$; (**b**) the voltage fluctuation reduces the resonance frequency when $V_{ac}=0.1\;V$.

As shown in Figure 6a, for $V_{ac}<0.171\;V$, the peak frequency increases with driving strength, but for the higher driving voltages, frequency stabilization occurs. Figure 9 shows the effect of voltage fluctuation on the dynamic behavior of a coupled resonance system when $V_{ac}<0.171\;V$ and $V_{ac}>0.171\;V$. The voltage noise causes the fluctuation of the amplitude–frequency response curve. It was noted that voltage noise has little effect on the peak frequency of the system when $V_{dc}=8\;V$, $V_{ac}=0.3\;V$, as shown in Figure 9a. However, the phenomenon of frequency stabilization disappears, and voltage noise greatly reduces the peak frequency when $V_{dc}=8\;V$, $V_{ac}=0.1\;V$, as shown in Figure 9b. Therefore, the frequency stabilization proposed in this paper can overcome the influence of voltage fluctuation on bifurcation frequency and improve the robustness of the sensor. This phenomenon can be explained as follows: When frequency stabilization occurs, the peak frequency is determined by the natural frequency of the cantilever beam, and the voltage fluctuation does not affect the peak frequency. As the driving voltage decreases, the frequency stabilization disappears, and the peak frequency is determined by the driving voltage. Thus, voltage fluctuation has an important impact on the peak frequency.

## 5. Parameter Identification Based on Frequency Stabilization and Bifurcation Jumping Behavior

The added mass can change the natural frequency of the system. Thus, researchers can identify the mass by measuring the resonance frequency of the system. The bifurcation-based mass sensing method greatly improves the sensitivity due to the sharpness of amplitude transition in a nonlinear regime [42,43,44]. However, the nonlinearity in flexural microbeam results in a dependence of bifurcation frequency on amplitude. In this section, we present an effort to explore the exploitation of frequency stabilization and bifurcation jump in a MEMS to realize a novel method for mass detection.

**Figure 10:**
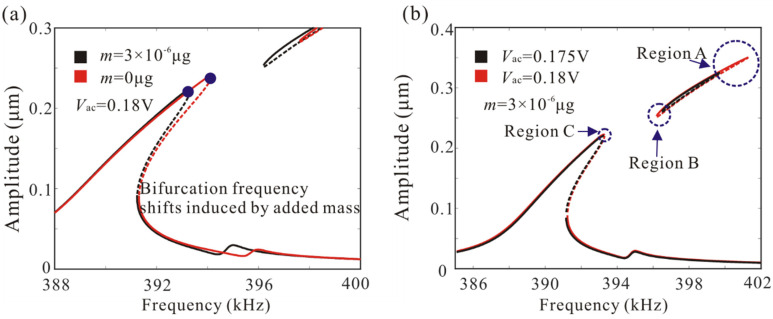
(**a**,**b**) Effect of added mass and AC voltage on the dynamic behavior of a coupled resonance system.

Figure 10 shows the variation of the frequency response curves versus drive voltage and added mass when *c_n_* = 0.04. It was noted that the bifurcation frequency of the resonator is reduced due to the added mass. In addition, the amplitude–frequency response curves under different driving voltages are given, as shown in Figure 10b. The result shows that the driving voltage mainly affects the bifurcation frequency in region A. However, the bifurcation frequency in region C is almost unaffected by AC voltage. Thus, the bifurcation frequency in region C is used to realize mass detection, which can improve the robustness of the sensor.

**Figure 11:**
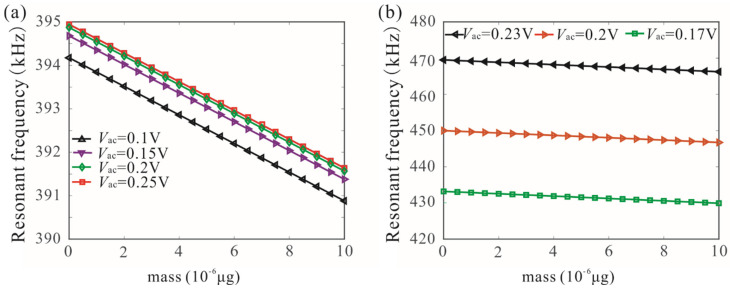
Variation of the bifurcation frequency of mechanically coupled microbeam resonators with different mass and AC voltage when *c_n_* = 0.02, $V_{dc}=8\;V$, and $L^{\prime }=59.2\;\mu m$. Here, (**a**) represents coupled resonant structure (the resonant frequency changes slightly when the voltage exceeds the critical value), and (**b**) represents the single-degree-of-freedom resonant structure.

**Figure 12:**
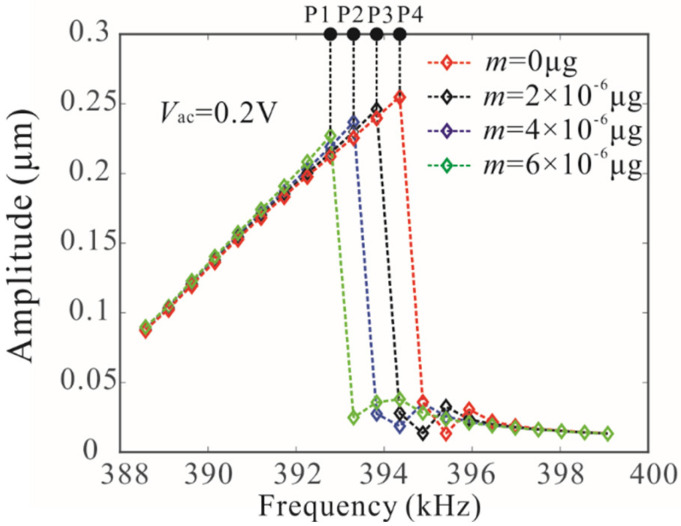
Amplitude resonance curves of microbeam 1 with different added mass. P1, P2, P3, and P4 represent resonance peak frequencies.

The variation of the bifurcation frequency of the coupled microbeam resonators with various values of the AC voltages and added mass was also studied, as shown in Figure 11. The bifurcation frequency decreases linearly with the increase of added mass. When the drive voltage is lower than the critical voltage of frequency stabilization, the voltage variation has obvious influence on the bifurcation frequency. With the increase of driving voltage, the influence of AC voltage on bifurcation frequency is very weak, as shown in Figure 11a. Similarly, we study the dynamic behavior of the single-degree-of-freedom system and found that voltage variation has a great influence on the bifurcation frequency. Here, we used bifurcation frequency, which is independent of the driving voltage under the condition of frequency stabilization, to realize mass detection. Amplitude resonance curves of microbeam 1 with a different added mass are shown in Figure 12.

Then, we try to derive a nonlinear parameter identification formula based on frequency stabilization and amplitude jump behavior. If the quality factor of the system is high enough, the bifurcation frequency of the system can be assumed as $\Omega \approx \omega_{2}/\sqrt{1+\eta^{\prime }}$ by Equation (20). Then, we can obtain

(24) $$\eta^{\prime }=\frac{\Omega_{1}^{2}-\Omega_{2}^{2}}{\Omega_{2}^{2}}$$

By dimensional transformation, the parameter identification formula is obtained:

(25) $$m=\frac{\Omega_{1}^{2}-\Omega_{2}^{2}}{\Omega_{2}^{2}\phi_{2}^{2}(L_{2}/L)}\rho AL$$

Following this, numerical studies are introduced to prove the mass detection method. P1, P2, P3, and P4 represent resonance frequencies when *m* = 6 × 10^−6^ µg, *m* = 4 × 10^−6^ µg, *m* = 2 × 10^−6^ µg and *m* = 0 µg in Figure 12. Through Equation (25), the mass detection results are obtained in Table 2. The results show that the parameter identification method presented in this paper can accurately identify the mass. However, as the damping increases, the error of mass detection increases gradually. In order to explain this phenomenon, we propose the error function of mass detection. In fact, we ignore the effect of added mass on the last term of Equation (20) and use Equation (24) to obtain an approximate mass.

Thus, the error function can be defined by Equations (20) and (24).

(26) $$\begin{array}{c} {\eta^{\prime }}_{\mathrm{error}}=\frac{c_{n}}{\omega_{2}\sqrt{1+\eta^{\prime }}}\sqrt{\frac{(1+\eta^{\prime })\kappa^{2}\omega_{1}/c_{n}\omega_{2}^{2}}{\sqrt{\lambda f^{2}/(\omega_{2}/\sqrt{1+\eta^{\prime }}-\omega_{1})\omega_{1}}-c_{n}\omega_{1}}-1}\\ -\frac{(1+\eta^{\prime })c_{n}}{\omega_{2}}\sqrt{\frac{\kappa^{2}\omega_{1}/c_{n}\omega_{2}^{2}}{\sqrt{\lambda f^{2}/(\omega_{2}-\omega_{1})\omega_{1}}-c_{n}\omega_{1}}-1} \end{array}$$

Substituting Equation (26) into Equation (24) yields the following:

(27) $$e=\left|\frac{{\eta^{\prime }}_{\mathrm{error}}}{\eta^{\prime }}\right|\times 100\%$$

**Figure 13:**
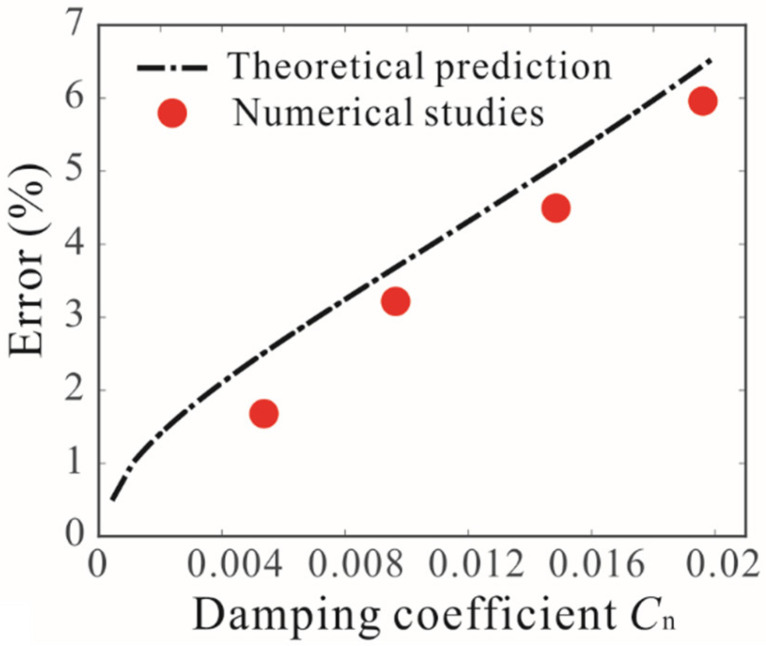
Variation of detection error under different quality factors when $V_{dc}=8\;V$, $V_{ac}=0.2\;V$.

Figure 13 shows the variation of the detection error under different quality factors. It was found that the damping is crucial to the accuracy of mass detection. When the damping is very small, the error function is negligible. As the damping increases, the value of the error function increases. The theoretical prediction results are obtained by Equation (27), and the numerical studies are obtained by long-time integration. It was found that they agree with each other. Thus, the quality factor of the resonator is the key to improving the accuracy of mass detection.

The parameter identification method solves the influence of voltage fluctuation on bifurcation frequency and improves the resolution of the sensor by using frequency stabilization and the bifurcation jump phenomenon, which is beneficial to the development of the nonlinear sensor. The added mass can be detected without considering the effect of the driving voltage in the nonlinear regime. This method is suitable for the coupled resonant structure with frequency stabilization. Improving quality factor is the key to improving the accuracy of sensor. It should be noted that this method is not applicable to the single-degree-of-freedom nonlinear structure. Its natural frequency is determined by the added mass and the driving voltage together, and the influence of the driving force needs to be considered. In addition, advantages and disadvantages of recently developed sensors are given in Table 3.

## 6. Conclusions

In the present work, a mechanically coupled resonant structure was designed to realize frequency stabilization by coupling a clamped–clamped microbeam and a cantilever microbeam. The nonlinear dynamic behaviors of a mechanically coupled resonant structure due to added mass were investigated. The following conclusions can be summarized.

(1) The physical conditions for frequency stabilization were presented when operating a MEMS in a nonlinear regime. It was found that improving quality factor is the key to achieving frequency stabilization. When the quality factor of the system is high enough, the effect of the driving voltage on the peak frequency can be ignored.
(2) Considering the voltage fluctuation, we analyzed the stochastic dynamic behavior of the coupled resonator under different driving voltages, and it was proven that the coupled resonator can overcome the influence of voltage noise on resonance frequency in the nonlinear regime and improve the robustness of the sensor.
(3) A very simple parameter identification formula was proposed to establish the relationship between resonance frequency and detection mass. Bifurcation and frequency stabilization were introduced to improve the accuracy and sensitivity of the sensor. The numerical results show that the mass identification method based on bifurcation frequency stabilization can make mass sensors work in a nonlinear vibration range and avoid resonance frequency shifts caused by driving voltage. The detection error function is presented to explain the difference between the detection results and the real results. It was noted that the mass detection method requires the MEMS resonator to have a high enough quality factor.

It is worth mentioning that the proposed concepts of coupled resonance sensors discussed in this paper need to be investigated for their stability to external disturbances. Periodic saddle bifurcation occurs near the resonance peak frequency. Hence, the stability of operation prior to mass detection must be ensured to prevent accidental bifurcation jump phenomenon due to noises or disturbances. This can be studied by conducting a global dynamic analysis to track the basin of attraction of the stable solution. In addition, subsequent experimental research can be carried out based on the above theoretical results on threshold expression and robustness analysis.

## Figures and Tables

**Table 1:** Mass sensor parameters and physical properties [48].

Physical Parameter (Units)	Value
Length of microbeam 1, *L* (µm)	150
Length of microbeam 3, *R* (µm)	10
Thickness of all microbeams, *h* (µm)	1
Widths of microbeams 1 and 2, *b* (µm)	10
Width of microbeam 3, $b^{\prime }$ (µm)	1
Position of the coupling beam, *L*_1_ (µm)	5
Mass adsorption position, *L*_2_ (µm)	50
Gap between the electrodes, *d* (µm)	1.5
Density of the electrode material, *ρ* (kg/m^3^)	2300
Young’s Modulus, *E* (GPa)	169
Dielectric constant of the medium, $\varepsilon_{0}$	8.85 × 10^−12^

**Table 2:** Nine groups of mass detection results when $V_{dc}=8\;V$, $V_{ac}=0.2\;V$.

Number	The True Mass *m* (10^−6^ μg)	Damping Coefficient *c_n_*	Identification Results *m* (10^−6^ μg)	The Ratio between the Analyte and the Sensor Mass	Error
1	2	0.02	1.884	0.04%	5.8%
2	4	0.02	3.768	0.08%	5.81%
3	6	0.02	5.647	0.12%	5.89%
4	2	0.01	1.939	0.04%	3.05%
5	4	0.01	3.876	0.08%	3.1%
6	6	0.01	5.813	0.12%	3.12%
7	2	0.005	1.976	0.04%	1.22%
8	4	0.005	3.95	0.08%	1.24%
9	6	0.005	5.922	0.12%	1.3%

**Table 3:** Comparison of advantages and disadvantages of recently developed sensors.

Sensor	Detection Principle	Nonlinear Factor	Advantages	Disadvantages
Mass sensor reported by Ekinci et al. [50]	The resonance frequency shifts induced by the added mass	Ignoring nonlinearity	The operation is simple	The influence of nonlinear factors is not considered
Accelerometer sensor reported by Peng et al. [51]	A change of modal amplitude ratio due to the mode localization	Ignoring nonlinearity	The sensitivity of the sensor is improved	The influence of nonlinear factors is not considered
Mass sensor reported by Nguyen et al. [44]	The bifurcation jumping induced by the added mass	Utilizing nonlinearity	Nonlinear jump can improve the sensitivity and resolution	Nonlinearity results in dependence of bifurcation frequency on amplitude
This work	The bifurcation frequency shifts induced by the added mass	Utilizing nonlinear coupled mode vibration	The dependence of bifurcation frequency on driving voltage is solved	High quality factors are needed

## Appendix A

To simplify Equations (1) and (2), nondimensional variables are introduced:

$$w_{1}=\frac{{\hat{w}}_{1}}{d},\;w_{2}=\frac{{\hat{w}}_{2}}{d},\;x=\frac{\hat{x}}{L},\;t=\hat{t}\sqrt{\frac{EI}{\rho AL^{4}}}$$

Then, Equations (1) and (2) can be written as the following nondimensional equations:

(A1) $$\begin{array}{l} \frac{\partial^{2}w_{1}}{\partial t^{2}}+w_{1}^{iv}+c_{1n}\frac{\partial w_{1}}{\partial t}-(\alpha_{1}\int_{0}^{1}{w_{1}^{\prime }}^{2}dx)w_{1}^{\prime \prime }\\ =\alpha_{2}\frac{V_{dc}^{2}}{{\left(1-w_{1}\right)}^{2}}-\alpha_{2}\frac{V_{dc}^{2}}{{\left(1+w_{1}\right)}^{2}}+\alpha_{2}\frac{2V_{dc}V_{ac}\cos\Omega t+{\left(V_{ac}\cos\Omega t\right)}^{2}}{{\left(1-w_{1}\right)}^{2}}+k^{\prime }(w_{2}-w_{1})\delta (x-l_{1}) \end{array}$$

(A2) $$\left(1+\eta )\frac{\partial^{2}w_{2}}{\partial t^{2}}+w_{2}^{iv}+c_{2n}\frac{\partial w_{2}}{\partial t}=k^{\prime }(w_{1}-w_{2})\delta (x-l_{1}\right)$$

with the following boundary conditions:

$$w_{1}(0,t)={w^{\prime }}_{1}(0,t)=w_{1}(1,t)={w^{\prime }}_{1}(1,t)=0$$

$$w_{2}(0,t)={w^{\prime }}_{2}(0,t)={w^{\prime \prime }}_{2}(l^{\prime },t)={w^{\prime \prime \prime }}_{2}(l^{\prime },t)=0$$

The parameters appearing in Equations (A1) and (A2) are as follows:

$$\alpha_{1}=6\times {\left(\frac{d}{h}\right)}^{2},\;\alpha_{2}=\frac{6\varepsilon_{0}L^{4}}{Ed^{3}h^{3}},\;\eta =\frac{\delta (x-L_{2}/L)m}{\rho AL},\;k^{\prime }=\frac{12L^{3}k}{Ebh^{3}},\;l_{1}=\frac{L_{1}}{L},\;l^{\prime }=\frac{L^{\prime }}{L}$$

Li et al. [48] found that the initial point of the system was stable when the driving force was small. The electrostatic force can be written as a Taylor series near the origin.

(A3) $$\begin{array}{l} \alpha_{2}\frac{V_{dc}^{2}}{{\left(1-w_{1}\right)}^{2}}-\alpha_{2}\frac{V_{dc}^{2}}{{\left(1+w_{1}\right)}^{2}}+\alpha_{2}\frac{2V_{dc}V_{ac}\cos\Omega t+{\left(V_{ac}\cos\Omega t\right)}^{2}}{{\left(1-w_{1}\right)}^{2}}\\ =\alpha_{2}V_{dc}^{2}(4w_{1}+8w_{1}^{3}+12w_{1}^{5}+\ldots )+2\alpha_{2}V_{dc}V_{ac}\cos\Omega t(1+2w_{1}+3w_{1}^{2}+\ldots )\\ +{\left(V_{ac}\cos\Omega t\right)}^{2}(1+2w_{1}+3w_{1}^{2}+\ldots ) \end{array}$$

High-order nonlinearity, the parametric excitation term, and the square term of AC voltage can be ignored when the system oscillates slightly near the origin [52,53]. Equation (A1) can be rewritten as

(A4) $$\begin{array}{l} \frac{\partial^{2}w_{1}}{\partial t^{2}}+w_{1}^{iv}+c_{1n}\frac{\partial w_{1}}{\partial t}-(\alpha_{1}\int_{0}^{1}{w_{1}^{\prime }}^{2}dx)w_{1}^{\prime \prime }\\ =\alpha_{2}V_{dc}^{2}(4w_{1}+8w_{1}^{3})+2\alpha_{2}V_{dc}V_{ac}\cos\Omega t+k^{\prime }(w_{2}-w_{1})\delta (x-l_{1}) \end{array}$$

Here, the solutions of Equations (A2) and (A4) can be expressed as $w_{1}(x,t)=\sum_{i=1}^{\infty }u_{1,i}(t)\phi_{1,i}(x)$ and $w_{2}(x,t)=\sum_{i=1}^{\infty }u_{2,i}(t)\phi_{2,i}(x)$, where $\phi_{1,i}$ and $\phi_{2,i}$ are the *i*-th linear undamped mode shape of microbeams 1 and 2. Then, the linear undamped eigenvalue equations are obtained:

(A5) $$\begin{array}{l} \phi_{1,i}^{iv}=\beta_{1,i}^{2}\phi_{1,i}+4\alpha_{2}V_{dc}^{2}\phi_{1,i}\\ \phi_{1,i}(0)=\phi_{1,i}(1)={\phi^{\prime }}_{1,i}(0)={\phi^{\prime }}_{1,i}(1)=0 \end{array}$$

(A6) $$\begin{array}{l} \phi_{2,i}^{iv}=\beta_{2,i}^{2}\phi_{2,i}\\ \phi_{2,i}(0)=\phi_{2,i}^{\prime \prime }(l^{\prime })=\phi_{2,i}^{\prime }(0)=\phi_{2,i}^{\prime \prime \prime }(l^{\prime })=0 \end{array}$$

By substituting Equations (A5) and (A6) into the resulting Equations (A2) and (A4), multiplying by $\phi_{2,i}$, $\phi_{1,i}$, and integrating the outcome from *x*=0 to $l^{\prime }$, *x*=0 to 1 [48], we obtain

(A7) $$\begin{array}{l} \frac{d^{2}u_{1,n}}{dt^{2}}+c_{1n}\frac{du_{1,n}}{dt}+\beta_{1,n}^{2}u_{1,n}-\sum_{i,j,k=1}^{M}[\alpha_{1}\int_{0}^{1}\phi_{1,i}^{\prime }\phi_{1,j}^{\prime }dx\int_{0}^{1}\phi_{1,k}^{\prime \prime }\phi_{1,n}dx+8\alpha_{2}V_{dc}^{2}\int_{0}^{1}\phi_{1,i}\phi_{1,j}\phi_{1,k}\phi_{1,n}dx]u_{1,i}u_{1,j}u_{1,k}\\ =2\alpha_{2}V_{dc}V_{ac}\cos\Omega t\int_{0}^{1}\phi_{1,n}dx+k^{\prime }[u_{2,n}\phi_{2,n}(l_{1})\phi_{1,n}(l_{1})-u_{1,n}\phi_{1,n}^{2}(l_{1})] \end{array}$$

(A8) $$\left(1+\eta^{\prime })\frac{d^{2}u_{2,n}}{dt^{2}}+c_{2n}\frac{du_{2,n}}{dt}+\beta_{2,n}^{2}u_{2,n}=k^{\prime }[u_{1,n}\phi_{2,n}(l_{1})\phi_{1}(l_{1})-u_{2,n}\phi_{2,n}^{2}(l_{1})\right]$$

where $\eta^{\prime }=\phi_{2,n}^{2}(L_{2}/L)m/\rho AL$.

In this paper, we mainly focus on the coupled dynamic behaviors near the fundamental frequency. Thus, we take *n* = 1 and obtain two-degree-of-freedom dynamical equations.

(A9) $$\begin{array}{l} \frac{d^{2}u_{1}}{dt^{2}}+c_{1n}\frac{du_{1}}{dt}+\beta_{1}^{2}u_{1}-[\alpha_{1}\int_{0}^{1}{\phi_{1}^{\prime }}^{2}dx\int_{0}^{1}\phi_{1}^{\prime \prime }\phi_{1}dx+8\alpha_{2}V_{dc}^{2}\int_{0}^{1}\phi_{1}^{4}dx]u_{1}^{3}\\ =2\alpha_{2}V_{dc}V_{ac}\int_{0}^{1}\phi_{1}dx\cos\Omega t+k^{\prime }[u_{2}\phi_{2}(l_{1})\phi_{1}(l_{1})-u_{1}\phi_{1}^{2}(l_{1})] \end{array}$$

(A10) $$\left(1+\eta^{\prime })\frac{d^{2}u_{2}}{dt^{2}}+c_{2n}\frac{du_{2}}{dt}+\beta_{2}^{2}u_{2}=k^{\prime }[u_{1}\phi_{2}(l_{1})\phi_{1}(l_{1})-u_{2}\phi_{2}^{2}(l_{1})\right]$$

Here, to simplify the equation, $u_{1,1}$, $u_{2,1}$, $\phi_{1,1}$, $\phi_{2,1}$ $\beta_{1,1}$ and $\beta_{2,1}$ are replaced by $u_{1}$, $u_{2}$, $\phi_{1}$, $\phi_{2}$ $\beta_{1}$ and $\beta_{2}$.

## Appendix B

The response of the mechanically coupled resonant structure with small amplitude vibration is studied by using the method of multiple scales. Based on the perturbation principle, *ε* is introduced as a small nondimensional bookkeeping parameter [54]. Here, the electrostatic force term f=O(ε) is assumed. Meanwhile, the magnitude of the dissipative terms should be consistent with that of the external excitation. Then, Equation (10) can be rewritten as

(A11) $$\frac{d^{2}u_{1}}{dt^{2}}+\varepsilon c^{\prime }\frac{du_{1}}{dt}+\omega_{n}^{2}u_{1}+k_{1a}u_{1}^{3}=\varepsilon f\cos\Omega t$$

To describe the nearness of the primary resonance, a detuning parameter σ is introduced and defined by

(A12) $$\Omega =\omega_{n}+\varepsilon \sigma$$

The approximate solution of Equation (A11) can be written as

(A13) $$u_{1}=y_{1}(T_{0},T_{1})+\varepsilon y_{2}(T_{0},T_{1})$$

where $T_{n}=\varepsilon^{n}t$.

Substituting Equations (A12) and (A13) into Equation (A11) and equating coefficients of like powers of ε yields the following:

(A14) $$Ο(\varepsilon^{0}):D_{0}^{2}y_{1}+\omega_{n}^{2}y_{1}=0$$

(A15) $$Ο(\varepsilon^{1}):D_{0}^{2}y_{2}+\omega_{n}^{2}y_{2}=-2D_{0}D_{1}y_{1}-c^{\prime }D_{0}y_{1}-k_{1a}y_{1}^{3}+f\cos(\omega_{n}T_{0}+\sigma T_{1})$$

where $D_{n}=\frac{\partial }{\partial T_{n}}$.

The solution of Equation (A14) can be written as $y_{1}(T_{0},T_{1})=A(T_{1})e^{i\omega_{n}T_{0}}+\bar{A}(T_{1})e^{-i\omega_{n}T_{0}}$.

To eliminate the secular term, we obtain

(A16) $$-2i\omega_{n}\frac{\partial A}{\partial T_{1}}=0$$

Then, we express *A* in the polar form:

(A17) $$A=\frac{1}{2}ae^{i\theta }+\mathrm{cc}$$

Substituting Equations (A16) and (A17) into Equation (A15) and separating the imaginary and real parts yields the following:

(A18) $$\frac{da}{dT_{1}}=-\frac{1}{2}c^{\prime }a+\frac{1}{2}\frac{f}{\omega_{n}}\sin\varphi$$

(A19) $$a\frac{d\varphi }{dT_{1}}=\sigma a-\frac{\lambda }{\omega_{n}}a^{3}+\frac{1}{2}\frac{f}{\omega_{n}}\cos\varphi$$

where $\varphi =\sigma T_{1}-\theta$, $\lambda =3k_{1a}/8$.

## Appendix C

Taking the square root of both sides of Equation (19), we can obtain

(A20) $$\sqrt{\lambda f^{2}/(\Omega -\omega_{1})\omega_{1}}-c_{n}\omega_{1}=\frac{\kappa^{2}c_{n}\omega_{1}}{{\left(c_{n}\Omega \right)}^{2}+{\left(\omega_{2}^{2}-\Omega^{2}-\eta^{\prime }\Omega^{2}\right)}^{2}}$$

Then, the Equation (A20) can be rewritten as

(A21) $${\left(\omega_{2}^{2}-\Omega^{2}-\eta^{\prime }\Omega^{2}\right)}^{2}=\frac{\kappa^{2}c_{n}\omega_{1}}{\sqrt{\lambda f^{2}/(\Omega -\omega_{1})\omega_{1}}-c_{n}\omega_{1}}-{\left(c_{n}\Omega \right)}^{2}$$

From Equation (A21), peak frequency Ω can be obtained:

(A22) $$\Omega^{2}=\frac{\omega_{2}^{2}}{1+\eta^{\prime }}\pm \frac{1}{1+\eta^{\prime }}\sqrt{\frac{\kappa^{2}c_{n}\omega_{1}}{\sqrt{\lambda f^{2}/(\Omega -\omega_{1})\omega_{1}}-c_{n}\omega_{1}}-{\left(c_{n}\Omega \right)}^{2}}$$

We consider that the drive frequency is near the natural frequency of microbeam 2. Then, the drive frequency can be expressed as

(A23) $$\Omega =\frac{\omega_{2}}{\sqrt{1+\eta^{\prime }}}+\varepsilon$$

where ε represents the disturbance term ($\varepsilon <<\omega_{2}/\sqrt{1+\eta^{\prime }}$).

Equation (A23) can be rewritten as

(A24) $$\Omega^{2}=\frac{\omega_{2}^{2}}{1+\eta^{\prime }}+\frac{2\omega_{2}}{\sqrt{1+\eta^{\prime }}}\varepsilon$$

Substituting Equation (A24) into Equation (A22) yields the following:

(A25) $$\varepsilon =\pm \frac{1}{2\omega_{2}\sqrt{1+\eta^{\prime }}}\sqrt{\frac{\kappa^{2}c_{n}\omega_{1}}{\sqrt{\lambda f^{2}/(\Omega -\omega_{1})\omega_{1}}-c_{n}\omega_{1}}-{\left(c_{n}\Omega \right)}^{2}}$$

Then, substituting Equation (A25) into Equation (A23) yields the following:

(A26) $$\Omega =\frac{\omega_{2}}{\sqrt{1+\eta^{\prime }}}\pm \frac{1}{2\sqrt{1+\eta^{\prime }}\omega_{2}}\sqrt{\frac{\kappa^{2}c_{n}\omega_{1}}{\sqrt{\lambda f^{2}/(\Omega -\omega_{1})\omega_{1}}-c_{n}\omega_{1}}-{\left(c_{n}\Omega \right)}^{2}}$$

When the quality factor is sufficiently large, we can replace Ω on the right hand of Equation (A26) with $\omega_{2}/\sqrt{1+\eta^{\prime }}$ [16]. Finally, the expression of peak frequency can be obtained:

(A27) $$\Omega =\frac{\omega_{2}}{\sqrt{1+\eta^{\prime }}}\pm \frac{c_{n}}{2(1+\eta^{\prime })}\sqrt{\frac{(1+\eta^{\prime })\kappa^{2}\omega_{1}/c_{n}\omega_{2}^{2}}{\sqrt{\lambda f^{2}/(\omega_{2}/\sqrt{1+\eta^{\prime }}-\omega_{1})\omega_{1}}-c_{n}\omega_{1}}-1}$$

To prove our derivation, we calculated the numerical solutions of Equation (A20) and Equation (A27) with MATLAB, respectively. It was found that they agree with each other.
